# Pertinent Questions

**Published:** 1888-02-11

**Authors:** 


### PERTINENT QUESTIONS.

Are private nurses from well-known institutions, trained for such institutions at well-known hospitals, who have proved excellent nurses, to be considered unfit for "trained nurses registration" owing to their not remaining three years probationers? Should the strain of private nursing prove injurious to the health of any, would their returning to hospital work before having "served their time" unqualify them, provided they hold good testimonials from doctors and matron? Are there not as many really good nurses (practically and theoretically) not holding examination certificates as there are with them? Does the working for an examination place the theory first?

A Constant Reader.

